# The Home Sister of My Alma Mater

**Published:** 1918-01-19

**Authors:** 


### SOME HOSPITAL TYPES OF BRITISH NURSING.

### The Home Sister of my Alma Mater.

THE character sketch which I attempt is that of the home sister of my *Alma Mater*, a woman of sterling worth, still occupying the same position, whose influence does still and will always remain with me throughout my career.

She was first and foremost a nurse in the truest sense of the word, and one of whom Florence Nightingale might well be proud.

She was a charming hostess, and endeavoured to make the Nurses' Home into a real "home"—a thankless task, in truth, and often apparently little appreciated by those for whom she did so much. Though ofttimes discouraged by this lack of appreciation, she never faltered in her task. A straight, upright, good, and noble woman, quietly religious, always punctual, very methodical, and the acme of neatness—a little narrow-minded perhaps as judged by modern ideas but an excellent example. Her standard of professional etiquette was very high, and she was intolerant of slackness in any form. She had favourites, however, among her nurses, and was perhaps a wee bit intolerant of those whom she disliked, maybe not always quite just in this respect—her one failing. She was the soul of loyalty, and a great comfort to any who were in trouble, being always ready to listen either to woes or joys and give sympathy or advice, a woman to whom Matron could open her heart at times and be quite sure of a loyal and sympathetic listener when the trials of hospital were almost beyond endurance.

The new probationer was always cheered by a few words of advice and a little "motherliness" from Sister while being shown how to make up and put on her cap. She was proud of her profession and of her uniform, and woe betide the nurse who wore it untidily or made up her cap to suit her style of hairdressing after once being shown the correct way!

Before being called to take up the duties of home sister she was sister of a male surgical ward, adored by her patients, but somewhat feared by her nurses. She always regretted being called upon to give up her ward, and was never quite so happy in her new sphere. Fortunate, indeed, were the nurses who worked in her ward, for they gained a groundwork of good knowledge which could never be forgotten and would serve them in good stead throughout their whole career. She was an excellent teacher, and her clinics in the ward—later her classes in the home—were looked forward to with real pleasure. She was satisfied with nothing but the best, full of encouragement for those who tried to do well, but very impatient with the slacker.

May Sister's health be long spared to enable her to carry on her good and zealous work, so that her influence will radiate through generations of nurses yet to pass through the school!

SISTER.

☛ **Previous articles appeared on Oct. 27, Dec. 1 and Dec. 22.**

